# The clinical characteristics and molecular mechanism of pituitary adenoma associated with meningioma

**DOI:** 10.1186/s12967-019-2103-0

**Published:** 2019-10-29

**Authors:** Haibo Zhu, Yazhou Miao, Yutao Shen, Jing Guo, Weiyan Xie, Sida Zhao, Wei Dong, Yazhuo Zhang, Chuzhong Li

**Affiliations:** 1 Beijing Neurosurgical Institute, Capital Medical University, No. 119, South Fourth Ring West Road, Fengtai District, Beijing, 100070 China; 2 Department of Neurosurgery, Beijing Tiantan Hospital affiliated to Capital Medical University, No. 119, South Fourth Ring West Road, Fengtai District, Beijing, 100070 China; 3 Beijing Institute for Brain Disorders Brain Tumor Center, No. 119, South Fourth Ring West Road, Fengtai District, Beijing, 100070 China; 4 China National Clinical Research Center for Neurological Diseases, No. 119, South Fourth Ring West Road, Fengtai District, Beijing, 100070 China

**Keywords:** Clinical characteristics, Molecular mechanism, MEN1, PAM, mTOR

## Abstract

**Background:**

Pituitary adenoma and meningioma are the most common benign tumors in the central nervous system. Pituitary adenoma associated with meningioma (PAM) is a rare disease and the clinical features and mechanisms of PAM are unclear.

**Methods:**

We summarized the clinical data of 57 PAM patients and compared with sporadic pituitary adenoma (SPA) and sporadic meningioma (SM). 5 pituitary adenomas of PAM and 5 SPAs were performed ceRNA microarray. qRT-PCR, Western Blot, siMEN1 and rapamycin inhibition experiment were validated for ceRNA microarray.

**Results:**

Clinical variable analyses revealed that significant correlations between PAM and female sex as well as older age when compared with SPA and significant correlations between PAM and transitional meningioma as well as older age when compared with SM. Additionally, the characteristics of PAM were significantly different for MEN1 patients. Functional experiments showed lower expression of MEN1 can upregulate mTOR signaling, in accordance with the result of ceRNA microarray. Rapamycin treatment promotes apoptosis in primary pituitary adenoma and meningioma cells of PAM.

**Conclusions:**

MEN1 plays an important role in PAM by upregulating mTOR signaling pathway. Rapamycin represents a potential therapeutic strategy for PAM in the future.

## Background

Pituitary adenoma and meningioma are the most common benign tumors in the central nervous system (CNS); pituitary adenomas represent a heterogeneous group of extra-axial neoplasms that collectively comprise approximately 13% of all intracranial tumors with an incidence of approximately 3 per 100,000 [1, 2]. Meningiomas are generally slow-growing tumors derived from the arachnoid membrane surrounding the central nervous system and they are among the most common intracranial tumors, with an overall incidence of 6 per 100,000 (15–25% of all brain tumors) and a 2:1 female to male ratio [3–6]. PAM is a rare clinical situation, and there were only 33 cases described before 2017 [7]. The precise cause for the development of PAM remains unknown. There are three possible explanations for PAM, including chance occurrence, environmental influence, or genetic predisposition. Currently there are no known epidemiological or well-characterized genetic associations between meningioma and pituitary adenoma.

Multiple endocrine neoplasia type 1 (MEN1) is an autosomal dominant disease caused by germline MEN1 mutations that leads to the development of multi-focal neoplastic endocrine lesions of the parathyroid glands, endocrine pancreas, duodenum, anterior pituitary, and, less commonly, stomach, adrenal cortex, thymus, and lungs [8–10]. In addition, various non-endocrine lesions may occur in the skin, CNS, and soft tissues. Asgharian et al. [11] reported that meningioma may be a component tumor of MEN1, and it is believed that alterations in the MEN1 gene may participates in its pathogenesis.

Hyperactivation of the PI3K/AKT/mTOR signaling pathway is found in many types of human cancers, and play key roles in regulating cell growth and tumorigenesis [12, 13]. Pachow et al. [14] reported that mTOR activation plays an important role in brain tumor pathogenesis and growth, including sporadic and syndromic brain tumors. Mutations in negative regulators of the mTOR pathway, such as PTEN, TSC1/TSC2 and NF1 are important for the tumorigenesis of familial cancer predisposition syndromes. Li et al. [15] reported that the mTOR pathway was related to the tumorigenesis of gonadotrophin adenoma. Meningioma samples have also been shown to express high levels of mTORC1 and S6K, implicating mTORC1 as a relevant signaling pathway in meningiomas [16]. In the present study, we found that lower expression of MEN1 play an important role in PAM by upregulating the mTOR signaling pathway. Rapamycin represents a potential therapeutic strategy for PAM in the future.

## Materials and methods

### Patients

We retrospectively reviewed pituitary adenoma patients in Beijing Tiantan Hospital from January 1, 2005 to December 31, 2017. All patients were classified according to preoperative images, including hormone, plain and enhanced head MRI, thin layer skull base CT scanning and three-dimensional reconstruction. Patients who suffered from meningioma and pituitary adenoma simultaneously or successively were included in this study. The present study was conducted in accordance with established ethical guidelines as outlined in the Declaration of Helsinki. We obtained written informed consent from all participants, and the Ethics Committee of Beijing Tiantan Hospital approved this study.

### ceRNA microarrays and construction of pathway act network

ceRNA means competing endogenous RNA. ceRNA microarray includes mRNA, lncRNA and circRNA. 5 pituitary adenomas of PAM and 5 SPAs were performed ceRNA microarray (Additional file 1: Table S1). Total RNA was extracted and purified using a mirVana™ miRNA Isolation Kit without phenol (Cat # AM1561, Ambion, Austin, TX, US). RNA samples from each group were then used to generate fluorescence-labeled cRNA targets for the SBC human ceRNA array V1.0 (4 × 180 K). The labeled cRNA targets were then hybridized with the slides. After hybridization, the slides were scanned on an Agilent Microarray Scanner (Agilent Technologies, Santa Clara, CA, US). The data were extracted with Feature Extraction software 10.7 (Agilent Technologies, Santa Clara, CA, US). Raw data were normalized by the Quantile algorithm using the limma package of the R program. The microarray experiments were performed according to the protocol of Agilent Technologies, Inc. at Shanghai Biotechnology Corporation. Ratios were calculated between the PAM and SPA. Then, differentially expressed genes were identified by using the t-test with a cut-off criteria of P < 0.05 and fold-change > 2 or < 0.5.

The selected mRNAs were grouped in functional categories based on the Gene Ontology database (GO: http://www.geneontology.org/). We identified the significant pathways of the differentially expressed genes by using IPA software (http://www.ingenuity.com). We used the software Cytoscape software (V2.8.0) (http://www.cytoscape.org) to construct a pathway act network for graphical representations of central pathways using the genes enriched in the significant canonical pathways of IPA (P < 0.05).

### Quantitative real-time PCR validation

Total RNA was extracted using TRIzol reagent (Invitrogen, USA) and then reversed transcribed using a HiFiScript gDNA Removal cDNA Synthesis Kit (CWBio, China) according to the manufacturer’s instructions. Subsequently, we performed qRT-PCR using SYBR Green assays in a total reaction volume of 10 μl which was performed on an ABI 7500 System (Applied Biosystems). GAPDH was used as a reference gene. For the quantitative analysis, expression levels were calculated based on CT values (corrected for GAPDH expression) according to the equation: 2^−∆CT^ [∆CT = CT (gene of interest) − CT (GAPDH)]. All qRT-PCR analyses were performed in triplicate. Student’s t-tests were applied, and a P-value < 0.05 was considered significant. The primer sequences are presented in Additional file 2: Table S2.

### Short interfering RNA transfection of HEK 293T cells

293T cells were authenticated by China Infrastructure of Cell Line Resource and tested negative for mycoplasma according to China Infrastructure of Cell Line Resource. Short interfering RNA (siRNA) against MEN1 (si-MEN1) and the negative control (sh-NC) were synthesized by RiboBio (Guangzhou, China) and employed (SiMEN1 CTACGACGGCATCTGCAAA). Exponentially growing HEK 293T cells (2 × 10^5^) were seeded onto 6-well plates overnight, and then transfected using Lipofectamine^®^3000 transfection reagent (Thermo Fisher Scientific, Massachusetts, USA) (final concentration: siRNA or negative control: 50 nM). The transfection efficiency was determined by qRT-PCR at 48 h after transfection. The protein extracted from transfected cells was collected to measure the level of MEN1and mTOR pathway genes by Western blot at 72 h after transfection.

### Western blot

Transfected 293T cells were lysed in nondenaturing lysis buffer (Applygen). For Western blotting, the protein samples (30 μg) were separated by 10% sodium dodecyl sulfate polyacrylamide gel electrophoresis and then transferred to polyvinylidene difluoride membranes. Different blots were incubated with antibodies against MEN1 (ab 92443, 1:5000, Abcam, USA), phosphor-AKT(1:1000, AF0016, Affinity, China), AKT(1:500, 10176-2-AP, Proteintech, USA), phospho-mTOR(1:500, AF3308, Affinity, China), mTOR(1:300, 20657-1-AP, Proteintech, USA) or vinculin(1:10,000, Abcam, USA), followed by incubation with secondary antibodies tagged with horseradish peroxidase (Santa Cruz Biotechnology). The blots were visualized by enhanced chemiluminescence, and densitometry was performed with an imaging apparatus (Amersham Imager 600, GE). Vinculin was used as a loading control.

### Isolation and culture of primary pituitary adenoma and meningioma cells of PAM

Before isolating primary pituitary adenoma and meningioma cells, all pituitary adenoma and meningioma biopsies were washed in PBS supplemented with P/S and 10% FBS in order to remove unhealthy tissues. After carefully washing, the remaining healthy tissues were cut into small pieces using refined scissors and dissociated into small cell clumps (10–50 cells/clump) with pre-chilled accutase. Then these small clumps of primary pituitary adenoma and meningioma cells were collected and re-suspended in cell culture medium: DMEM/F12 (Invitrogen), 1% Knock Out serum replacement (KSR, Invitrogen), N2 supplement (100X, Invitrogen), B27 supplement (100X, Invitrogen),1 mM l-Glutamine, 0.1 mM NEAA, 0.1 mM 2-ME with 4 ng/ml bFGF (R&D systems), and 10 ng/ml EGF (R&D systems). After evaluating the cell number and viability, primary pituitary adenoma and meningioma cells were seeded at 2 × 10^5^ cells/well (6 well plate) on Matrigel-embedded plate. The medium was replenished every 24 h for 5–7 days before passaging. Pituitary adenoma and meningioma cells can be passaged by accutase digestion and can be routinely maintained in cell culture medium for 5–10 passages.

### Apoptosis induction assays

For the human FAS;CD95 ELISA assay, pituitary adenoma cells were plated into 96 well plate at 2 × 10^4^ cells/well and treated with rapamycin at 0 nM, 0.5 nM, 1 nM, 2 nM. and Meningioma cells were plated into 96 well plate at 2 × 10^4^ cells/well and treated with rapamycin at 0 nM, 1 nM, 2 nM, 5 nM, 10 nM. Supernatants were collected at 48 h and 72 h for apoptosis detection. Apoptosis index was measured following the instruction of the human FAS;CD95 ELISA kit (KS4622, Keshun biotechnology).

For the annexin V Conjugates based assay, pituitary adenoma cells were plated into 96 well plate at 2 × 10^6^ cells/well and treated with rapamycin at 0.5 nM, 1 nM, 2 nM. Meningioma cells were plated into 96 well plate at 2 × 10^6^ cells/well and treated with rapamycin at 2 nM, 5 nM, 10 nM. Supernatants were collected at 48 h and 72 h for apoptosis detection. Cells were then treated with Annexin V Alexa Fluor 488 to identify apoptotic cells by flow cytometry.

### Statistical analysis

Statistical analysis was performed using SPSS software (version 20.0, IBM). The χ^2^ test was used to analyze metric variables. The two independent-samples t test was used to test ordinal variables. P-values of < 0.05 were defined as a significant difference.

## Results

### Clinical features

**Fig. 1:**
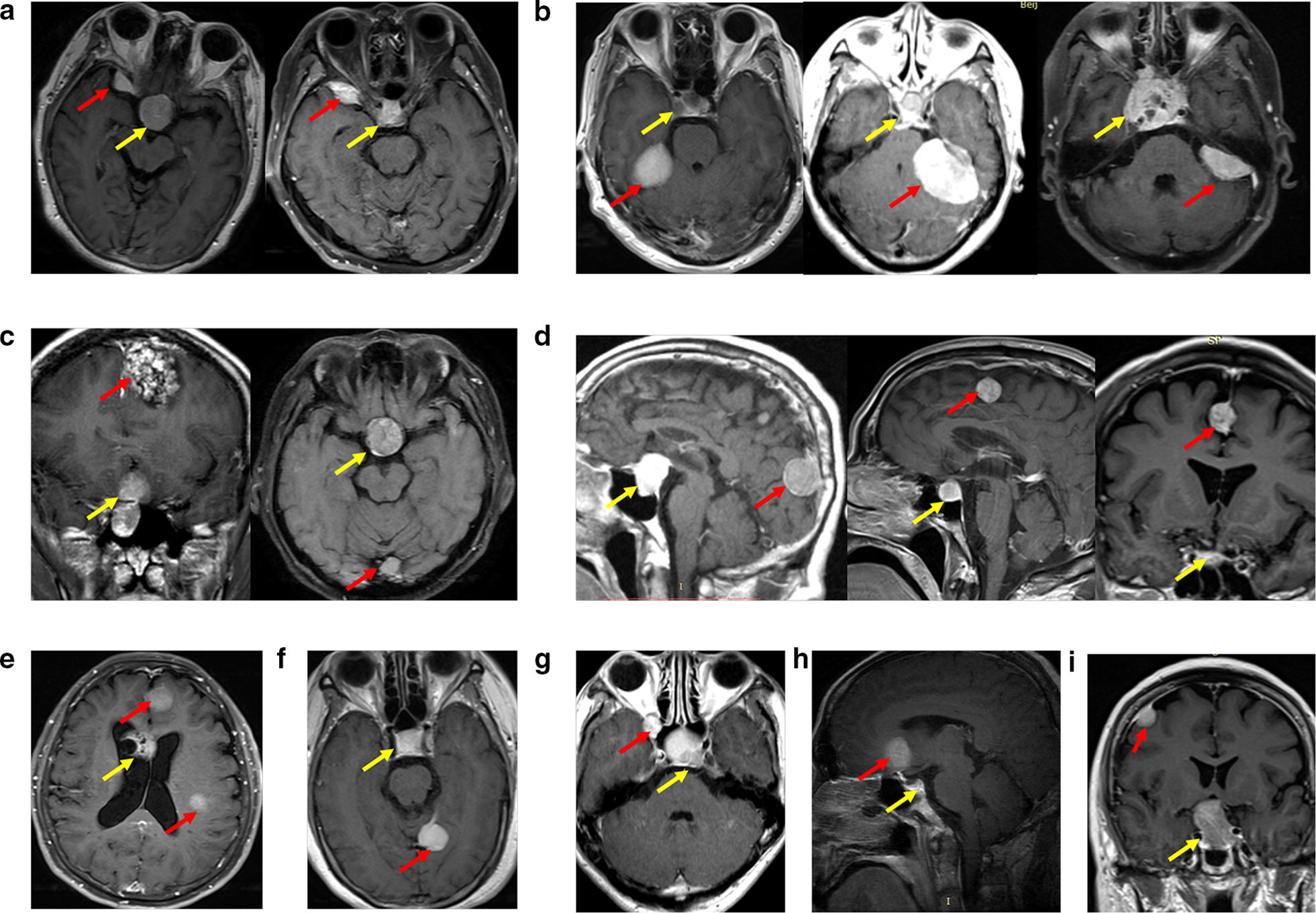
Pituitary adenoma associated with meningioma which located different anatomical region: **a** sphenoid ridge; **b** cerebellopontine angle; **c** parasagittal; **d** parafalcine; **e** multiple; **f** tentorium cerebelli; **g** orbital; **h** tuberculum sellae; **i** brain convex. Red arrow indicate meningioma and yellow arrow indicate pituitary adenoma

**Fig. 2:**
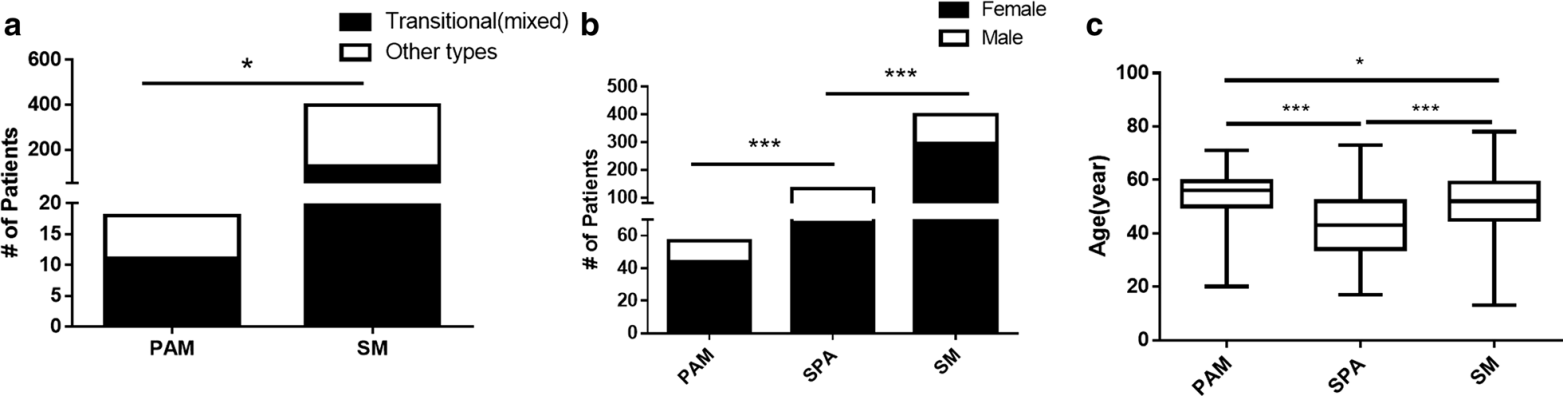
Clinical variable analyses between PAM and SPA, SM: **a** pathological difference between PAM and SM; **b** sex difference between PAM and SPA, SM; **c** age difference between PAM and SPA, SM. *P < 0.05, **P < 0.01, ***P < 0.001

A total of 8197 pituitary adenoma patients from January 1, 2005 to December 31, 2017, were retrospectively analyzed; of these, 57 (0.7%) patients met the criteria for PAM (Additional file 3: Table S3). The average age was 54.2 ± 9.8 years (ranging from 20 to 71 years), with 44 female and 13 male patients. In 55 PAM patients, 51 were diagnosed as pituitary adenoma and meningioma simultaneously, 4 were diagnosed with meningioma first, and 2 were diagnosed with pituitary adenoma first. All pituitary adenomas were resected, with 20 invasive, 34 non-invasive, and 3 not applicable, including 1 ACTH, 6 GH, 1 GH + PRL, 34 NFPA and 7 PRL cases. Meningiomas were located at different positions, including 17 brain convex, 12 parafalcine, 7 skull base, 6 tentorium cerebelli, 5 cerebellopontine angle, 4 multiple, 3 parasagittal, 2 orbital and 1 T9–10 cases (Fig. 1). 18 meningiomas were resected, and there were 11 transitional, 5 fibrous and 2 endothelial meningioma. A total of 134 SPAs and 399 SMs were involved in the present study for clinical variable statistical analysis (Additional file 4: Table S4). Clinical variable analyses between PAM and SM revealed significant correlations between PAM and transitional meningioma (P = 0.018) and older age (P = 0.05). Clinical variable analyses between PAM and SPA revealed that significant correlations of PAM with female sex (P = 0.0007) and older age (P < 0.0001) (Fig. 2).

### Identified differentially expressed mRNAs by transcriptome microarrays

**Fig. 3:**
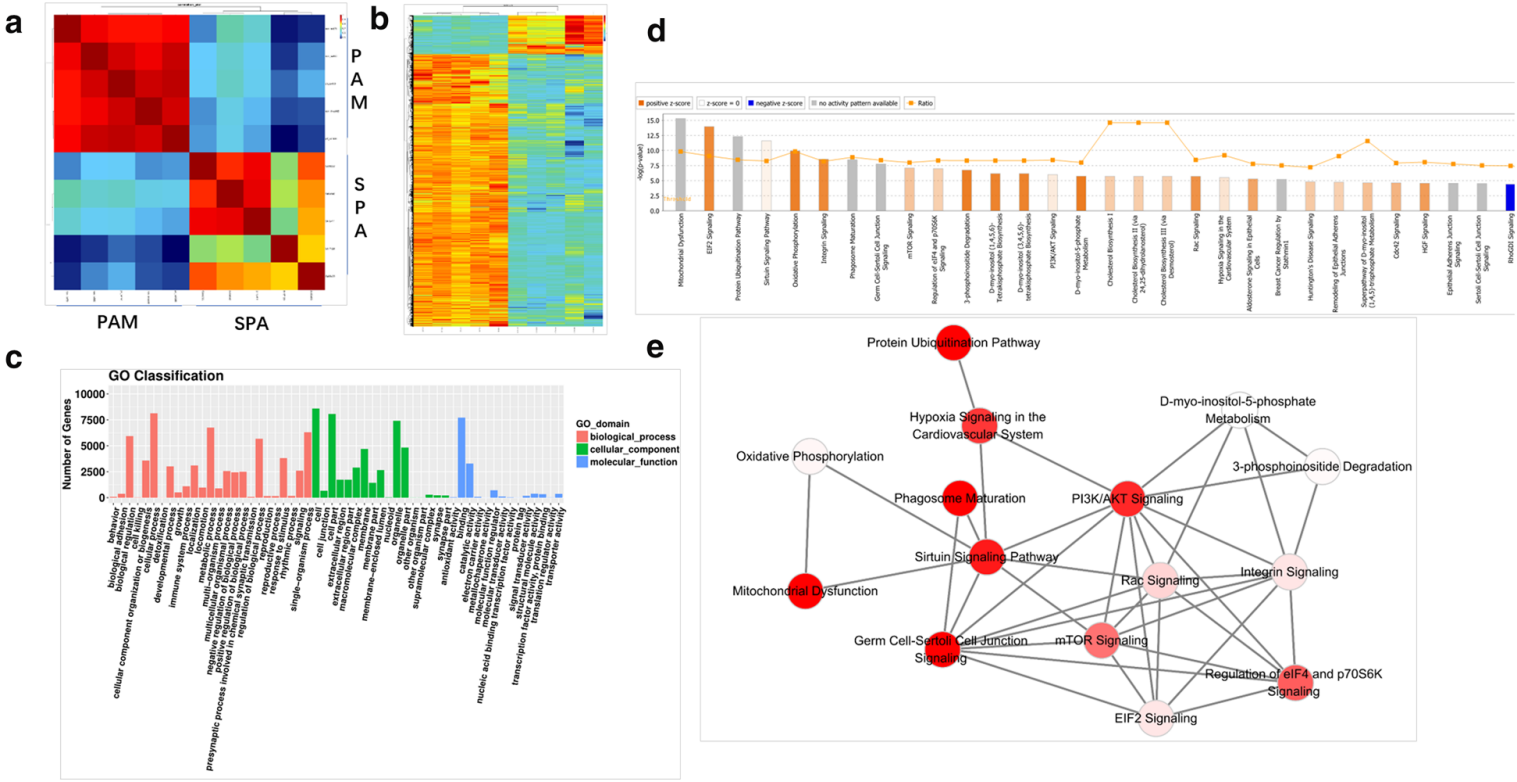
Correlation analysis and Cytoscape pathway act network by transcription microarray: **a** correlation plot showed the correlation between each sample. The brightness of the color represents the degree of correlation between the samples. Red indicates high correlation and blue indicates low correlation; **b** heat map shows the expression profiles of mRNAs between pituitary adenoma samples of PAM and SPA samples (FC > 2 or < 0.5 and P < 0.05), each row represents a single mRNA, and each column represents one sample. Red indicates high expression and green indicates low expression; **c** go annotation of differentially expressed mRNAs with the enrichment covering domains of biological processes, cellular components and molecular functions; **d** IPA pathway analysis of mRNAs enriched in the top thirty pathways according to the P-value; **e** Cytoscape pathway act network: Pathway act network according to the overlap of common differentially expressed molecules in top 15 significant canonical pathways. The node color is associated with pathway status. Red indicates that the signaling pathway is activated contrast to green indicates that the signaling pathway is suppressed. Grey indicates that the pathway is unpredicted

Compared with SPAs, 10188 mRNAs were significantly differentially expressed in pituitary adenomas of PAM, including 8918 upregulated and 1270 downregulated mRNAs (Additional file 5: Table S5). Correlation plot analysis showed that the correlation between the samples of the two groups was low and the correlation between the samples of each group was high (Fig. 3a). Hierarchical clustering showed that the expression patterns of the mRNAs between the two groups were distinguishable (Fig. 3b).

### GO and IPA analysis of differentially expressed mRNAs

GO analysis was performed to study the biological processes, cellular components and specific molecular functions of all differentially expressed mRNAs. We performed GO analysis of the mRNAs that were differentially expressed between the two groups. The results showed that the biological processes mainly involved the cellular process, metabolic process, regulation of biological process and single-organism process; the cellular components mainly involved the cell, cell part, and organelle; and the molecular functions mainly involved binding and catalytic activity (Fig. 3c). IPA pathway analysis targeting differentially expressed mRNAs (the top 30 pathways with the highest enrichment scores were selected) revealed that differentially expressed mRNAs were mainly involved in Mitochondrial Dysfunction, EIF2 Signaling, Protein Ubiquitination Pathway, Sirtuin Signaling Pathway, Oxidative Phosphorylation, Integrin Signaling, Phagosome Maturation, Germ Cell-Sertoli Cell Junction Signaling, mTOR Signaling, etc. (Fig. 3d).

### Cytoscape pathway act network

We used Cystoscope to construct a pathway act network according to the overlap of common differentially expressed molecules in the top 15 canonical pathways (Fig. 3e). The results showed that the protein ubiquitination pathway, mTOR signaling, PI3K/AKT signaling, Regulation of eIF4 and p70S6K signaling, Mitochondrial dysfunction, Sirtuin Signaling, Phagosome maturation, Germ cell-sertoli cell junction signaling pathway and Hypoxia signaling in the cardiovascular system were activated in the network. The mTOR signaling pathway was the core node in the pathway network. We selected the mTOR pathway for further verification.

### Validation of microarray data by qRT-PCR and Western Blot

**Fig. 4:**
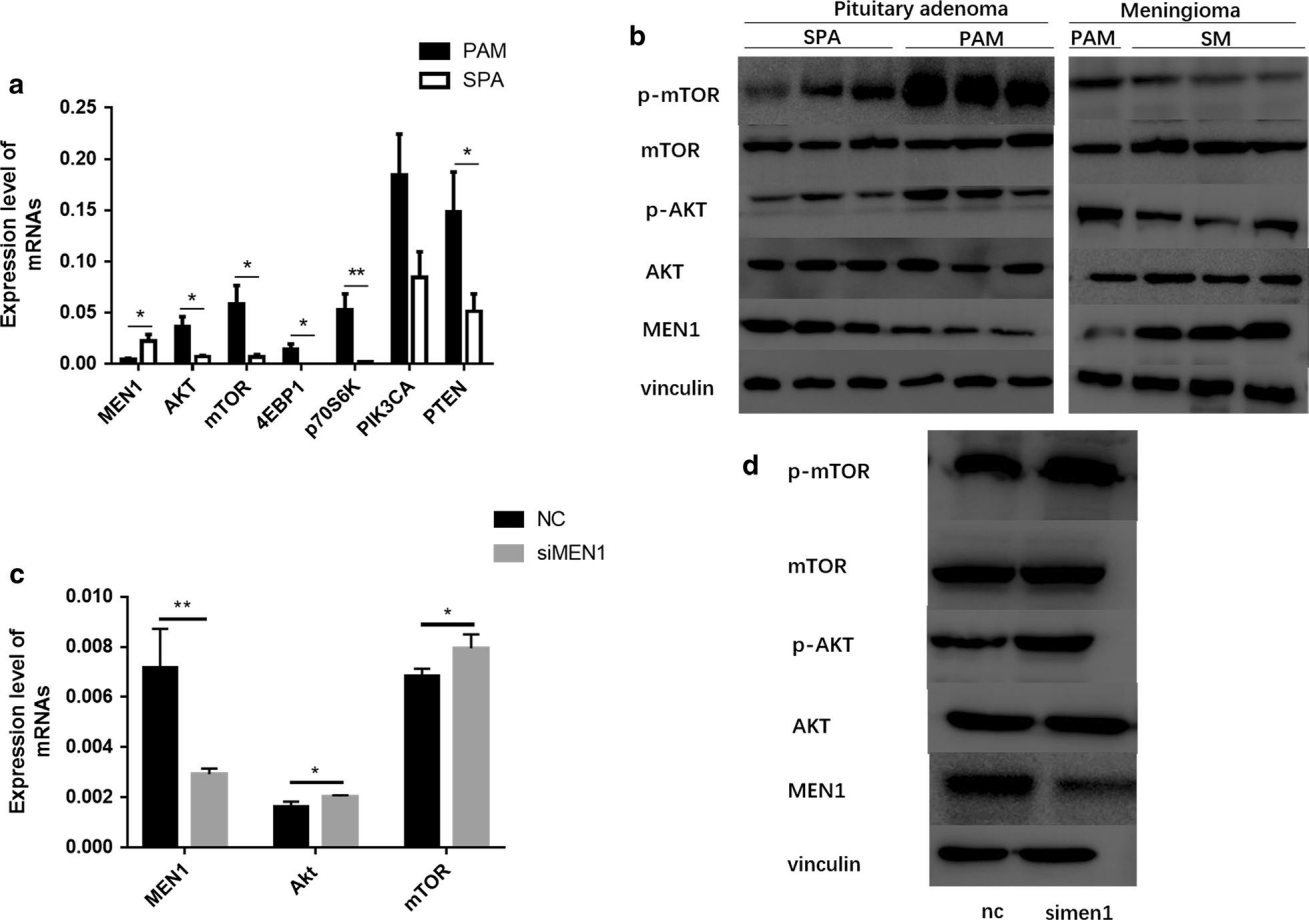
MEN1 play an important role in PAM through regulating mTOR signaling pathway: **a** qRT-PCR: the expression levels of 7 mRNAs; **b** Western blot analysis of MEN1, AKT, p-AKT, mTOR and p-mTOR in PAM, SPA and SM; **c** the expression levels of MEN1, Akt and mTOR after treatment with siMEN1 or NC in 293T cell; **d** Western blot analysisof MEN1, AKT, p-AKT, mTOR and p-mTOR after treatment with siMEN1 or NC in 293T cell. *P < 0.05, **P < 0.01, ***P < 0.001

Based on the bioinformatics prediction, we selected 7 mRNAs of the mTOR signaling pathway, and the results showed that the expression levels of MEN1, AKT, mTOR, 4E-BP1, p70S6K and PTEN were significantly different between the two groups (Fig. 4a). Compared with SPAs and SMs, the protein p-AKT (phosphorylated at Ser473), p-mTOR (phosphorylated at Ser2448) all showed increasing expression trend, and MEN1 showed reducing expression trend in pituitary adenoma and meningioma samples of PAM (Fig. 4b).

### siMEN1 promoted the expression of mTOR signaling pathway genes

The factors involved in the AKT/mTOR pathway have been known to participate in the tumorigenesis and tumor progression of pituitary adenoma and meningioma [15, 17, 18]. The interfering efficiency for siMEN1 was observed at 48 h after transfection. The gene expression levels of AKT and mTOR were significantly increased and the expression level of MEN1 was significantly reduced (Fig. 4c). When their total and phosphorylated protein levels were detected, the proteins p-AKT (phosphorylated at Ser473) and p-mTOR (phosphorylated at Ser2448) showed an increasing expression trend, and MEN1 showed a reducing expression trend (Fig. 4d). These results suggested that the low expression of MEN1 could activate the expression of factors involved in the mTOR signaling pathway.

### Rapamycin promotes apoptosis in primary pituitary adenoma and meningioma cells of PAM

**Fig. 5:**
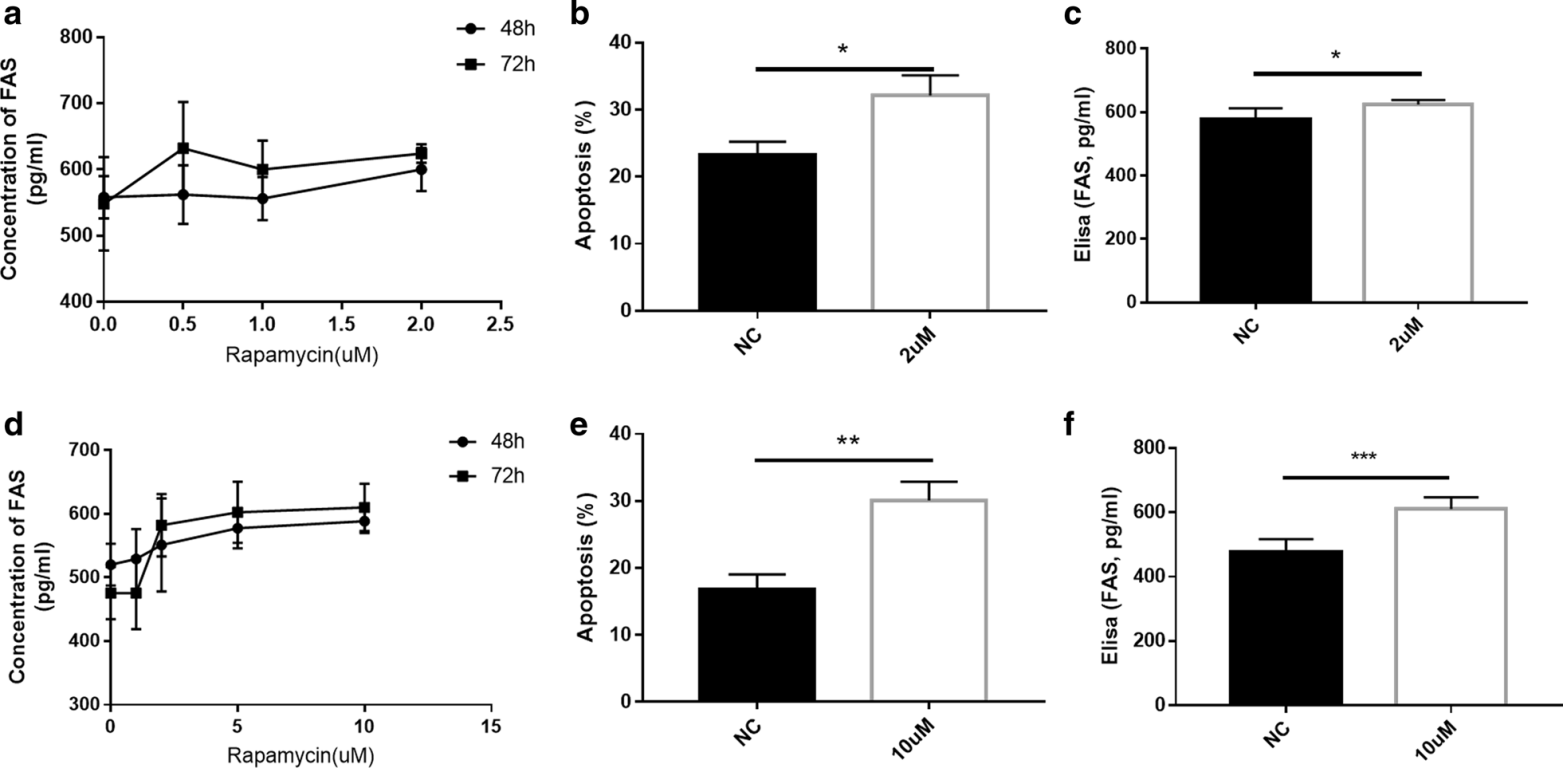
Rapamycin promotes apoptosis in primary pituitary adenoma and meningioma cells of PAM: **a** Flow cytometry revealing that the apoptosis rate of primary pituitary adenoma cells of PAM increased following treatment with rapamycin treatment in a dose-dependent manner for 48 h and 72 h; **b** Flow cytometry revealing that the apoptosis rate of primary pituitary adenoma cells increased following treatment with rapamycin 2 nM for 72 h; **c** Flow cytometry revealing that the FAS concentration of primary pituitary adenoma cells increased following treatment with rapamycin 2 nM for 72 h; **d** Flow cytometry revealing that the apoptosis rate of primary meningioma cells of PAM increased following treatment with rapamycin treatment in a dose-dependent manner for 48 h and 72 h; **e** Flow cytometry revealing that the apoptosis rate of primary meningioma cells increased following treatment with rapamycin 10 nM for 72 h; **f** Flow cytometry revealing that the FAS concentration of primary pituitary adenoma cells increased following treatment with rapamycin 10 nM for 72 h* P < 0.05, **P < 0.01, ***P < 0.001

Elisa analysis demonstrated that rapamycin upregulated FAS expression in a dose‑dependent manner in primary pituitary adenoma and meningioma cells of PAM (Fig. 5a, f). FITC-Annexin V/PI double staining was used to detect apoptosis in primary pituitary adenoma and meningioma cells of PAM following rapamycin treatment. Following treatment with 2 nM rapamycin in primary pituitary adenoma cells, the rate of apoptosis (P = 0.013) and the level of FAS (P = 0.029) significantly increased compared with the control (Fig. 5b–e). Following treatment with 10 nM rapamycin in primary meningioma cells, the rate of apoptosis (P = 0.003) and the level of FAS (P = 0.0006) significantly increased compared with the control (Fig. 5g–j). These results indicated that rapamycin treatment promotes apoptosis in primary pituitary adenoma and meningioma cells of PAM. In the future, rapamycin represent potential therapeutic strategy for PAM.

## Discussion

Pituitary adenoma and meningioma are the most common benign tumors in the CNS; there were only 33 PAMs described before 2017 [7]. We summarized 8197 pituitary adenomas in our hospital, and there were 57 PAMs, 5 pituitary adenoma associated with glioma and 5 pituitary adenoma associated with schwannoglioma in the present study. The incidence of meningioma between the normal population (6/100,000) and pituitary adenoma patients (7/1000) had a significant difference. A recent study reported that meningioma of PAM may be associated with GH and IGF-1 levels and radiology [19]. However, in the present study, there were only 6 GH patients and the 2 PAM patients who were diagnosed with pituitary adenoma first had no history of radiotherapy. There may be a common genetic mechanism leading to PAM.

The PI3K/AKT/mTOR signaling pathway plays a key role in regulating cell proliferation, cell growth, apoptosis and metabolism [20, 21]. Some studies reported that the PI3K/AKT/mTOR pathway plays an important role in pituitary adenoma and meningioma [15–18]. A recent study reported that both sporadic and syndromic brain tumors are related to hyperactivation of mTOR [14]. Li et al. reported that *MEN1*/Menin regulates milk protein synthesis through mTOR signaling in mammary epithelia cells. Mafficini et al. [22] reviewed that MEN1 can regulate mTOR pathway by inhibiting AKT in pancreatic neuroendocrine tumors. In the present study, we found that compared with SPA or SM, the expression of MEN1 was lower and the mTOR signaling pathway was hyperactivated in PAM patients. Therefore, lower expression of MEN1 plays an important role in PAM by regulating mTOR signaling pathway.

Inhibition of the mTOR signaling pathway has become an attractive target for human cancer therapy. Rapamycin is an mTOR inhibitor with potent immunosuppressive and antiproliferative effects [23]. Several clinical trials using mTOR inhibitors to treat brain tumors showed that the clinical value of single or combined treatment of primary or recurrent glioblastoma is unclear [24–26]. However, subependymal giant cell astrocytomas (SEGAs) are characterized by high expression levels of activated (phosphorylated) S6K, and these tumors are exquisitely responsive to treatment with the mTORC1 inhibitor everolimus [27–29]. Additionally, no pituitary adenoma and meningioma clinical studies have been reported so far, but a recent study showed that mTORC1 inhibitors suppress meningioma growth in mouse models; thus, meningiomas might represent a suitable target [16]. Cerovac et al. [30] reported that adjuvant treatment with a somatostatin analogue can sensitize pituitary tumor cells to the anti-proliferative effects of rapamycin. In the present study, we found that rapamycin treatment promotes apoptosis in primary cells of pituitary adenoma and meningioma of PAM. Therefore, these results indicated that rapamycin represent potential therapeutic strategy for PAM in the future.

## Conclusion

Taken together, There are significant correlations between PAM and female sex as well as older age when compared with SPA and significant correlations between PAM and transitional meningioma as well as older age when compared with SM.MEN1 plays an important role in PAM by upregulating mTOR signaling pathway. Rapamycin represents a potential therapeutic strategy for PAM in the future.

## Supplementary information

**Additional file 1: Table S1.** Clinical information of ceRNA microarray.

**Additional file 2: Table S2.** PCR primers of mRNAs used for qRT-PCR.

**Additional file 3: Table S3.** Clinical information of 57 PAMs.

**Additional file 4: Table S4.** Clinical information of SPA.

**Additional file 5: Table S5.** Different expression genes between PAM and SPA.

## Data Availability

The datasets during and/or analyzed during the current study available from the corresponding author on reasonable request.

## Abbreviations

PAM: pituitary adenoma associated with meningioma
SPA: sporadic pituitary adenoma
SM: sporadic meningioma
siRNA: small interfering RNA
GO: gene ontology
IPA: Ingenuity Pathway Analysis
ceRNA: competing endogenous RNA
MEN1: multiple endocrine neoplasia 1
CNS: central nervous system
FFPE: Formalin-Fixed and Paraffin-Embedded
CCF: cancer cell fraction
