# Supplementary material for: The clinical characteristics and molecular mechanism of pituitary adenoma associated with meningioma
Source: J Transl Med. 2019 Oct 29;17:354. doi: 10.1186/s12967-019-2103-0 (PMC6821033; doi:10.1186/s12967-019-2103-0)
Supplement: Supplementary file 1 — Additional file 1: Table S1. Clinical information of ceRNA microarray. [file 12967_2019_2103_MOESM1_ESM.docx]

**Table S1 Clinical information of ceRNA microarray**

| Group | sex | Age at  PA Dx | Age at Meningioma Dx | Attributable symptoms | Invasion | Volume(mm3) | Endocrine function | Meningioma location | Volume  (mm3) | Pathology | Associated pathologies |
| --- | --- | --- | --- | --- | --- | --- | --- | --- | --- | --- | --- |
| PAM | M | 65 | 65 | Physical findings | N | 5814 | NFPA | Left temporal | 500 | NA | NA |
| PAM | F | 67 | 67 | Decline of vision | N | 19320 | NFPA | Right sphenoid ridge | 2448 | NA | NA |
| PAM | F | 53 | 53 | Physical findings | N | 11475 | NFPA | Right frontal | 3744 | NA | NA |
| PAM | F | 54 | 54 | Decline of vision | N | 15708 | NFPA | Parafalcine | 3780 | NA | NA |
| PAM | M | 61 | 61 | Physical findings | N | 2925 | NFPA | Parafalcine | 1080 | NA | NA |
| SPA | F | 56 | N | Headache | Y | 11800 | NFPA | N | N | N | N |
| SPA | M | 48 | N | Headache | N | 3149 | NFPA | N | N | N | N |
| SPA | M | 42 | N | Decline of vision | N | 11664 | NFPA | N | N | N | N |
| SPA | F | 55 | N | Decline of vision | Y | 27892 | NFPA | N | N | N | N |
| SPA | M | 61 | N | Decline of vision | N | 13036 | NFPA | N | N | N | N |

**Abbreviation:** M, male, F, female, Y, yes, N, no, NA, not available.
