# Supplementary material for: The clinical characteristics and molecular mechanism of pituitary adenoma associated with meningioma
Source: J Transl Med. 2019 Oct 29;17:354. doi: 10.1186/s12967-019-2103-0 (PMC6821033; doi:10.1186/s12967-019-2103-0)
Supplement: Supplementary file 2 — Additional file 2: Table S2. PCR primers of mRNAs used for qRT-PCR. [file 12967_2019_2103_MOESM2_ESM.docx]

**Table S2 PCR primers of mRNAs used for qRT-PCR**

| Gene symbol | Gene type | Forward primer | Reverse primer |
| --- | --- | --- | --- |
| Akt1 | mRNA | 5‘CCCAGGTCACGTCGGAGACTGA3’ | 5‘GCTCGCTGTCCACACACTCCAT3’ |
| PTEN | mRNA | 5‘AATGTGAAGGTCTGAATGAGGG3’ | 5‘TAATGCCATTTTTCCATTTCCA3’ |
| PIK3CA | mRNA | 5‘GCTCCCACAAAGTAAAAAAAAAA3’ | 5‘GCAGAAGGAGAAAAAAAATCAAC3’ |
| TSC2 | mRNA | 5‘CGACCAGATCCCATCATACGAC3’ | 5‘CCGTGTACCTGTAGGAGCCATG3’ |
| mTOR | mRNA | 5‘TTCTCACAGACATTGGTCGGTA3’ | 5‘TGTTCTTCAGAATCTTGTTGGC3’ |
| 4EBP1 | mRNA | 5‘AGCCATCGTGTGGAGCACTACC3‘ | 5‘CCAACGCCTGCCCAGTATGAT3‘ |
| p70S6K | mRNA | 5‘TGGGTTCATCTATGTTTGTGTTTG3‘ | 5‘CATAGCACTCCTAATAAGGCAGC3‘ |
| MEN1 | mRNA | 5‘TGCCCCAGTCGTTAGAATATAGG3‘ | 5‘GAGGAGCTTGGGTTTCTAGGG3‘ |
