# Supplementary material for: The clinical characteristics and molecular mechanism of pituitary adenoma associated with meningioma
Source: J Transl Med. 2019 Oct 29;17:354. doi: 10.1186/s12967-019-2103-0 (PMC6821033; doi:10.1186/s12967-019-2103-0)
Supplement: Supplementary file 3 — Additional file 3: Table S3. Clinical information of 57 PAMs. [file 12967_2019_2103_MOESM3_ESM.docx]

**Table S3 Clinical information of 57 PAMs**

| NO. | Sex | Order of onset | Age at PA Dx | Age at Meningioma Dx | Attributable symptoms | Inva-  sion | Volume  (mm3) | Endocrine function | Meningioma location | Volume  (mm3) | Pathology | Associated pathologies |
| --- | --- | --- | --- | --- | --- | --- | --- | --- | --- | --- | --- | --- |
| 1 | F | Men | 50 | 44 | Decline of vision | N | 3264 | NFPA | Left frontal | 48000 | Transitional | NA |
| 2 | F | S | 66 | 59 | Decline of vision | Y | 112500 | NFPA | Tentorium cerebelli | NA | Fibrous | NA |
| 3 | M | S | 37 | 37 | Visual field defects | N | 26250 | NFPA | Obital apex | 1300 | NA | NA |
| 4 | F | S | 50 | 50 | Decline of vision | N | 9234 | NFPA | Left frontal | 280 | NA | NA |
| 5 | F | Men | 71 | 65 | Decline of vision | N | 1650 | NFPA | Tentorium cerebelli | 15625 | Transitional | NA |
| 6 | F | S | 44 | 42 | Menstrual disorder | NA | NA | GH | Left temporal | 16500 | Transitional | NA |
| 7 | F | Men | 62 | 54 | Decline of vision | N | 2160 | PRL | Right petroclival | NA | NA | Adrenal tumors |
| 8 | F | S | 46 | 46 | Amenorrhea | Y | 50568 | PRL | Left temporal occipital | 6000 | NA | NA |
| 9 | F | S | 20 | 20 | Menstrual disorder | Y | 15400 | PRL | Right parasagittal | 46332 | Endothelial | NA |
| 10 | F | S | 58 | 58 | Decline of vision | N | 11025 | NFPA | Left CPA | 2160 | NA | NA |
| 11 | F | S | 48 | 48 | Acromegaly | N | 6358 | GH | Right frontal | 90 | NA | NA |
| 12 | F | S | 45 | 45 | Amenorrhea | Y | 62640 | PRL | Left temporal | 1980 | NA | NA |
| 13 | M | S | 36 | 36 | Decline of vision | N | 5054 | PRL | Parafalcine | 1000 | NA | NA |
| 14 | F | S | 39 | 39 | Amenorrhea, Lactation | N | 2688 | NFPA | Parafalcine | 2016 | NA | NA |
| 15 | F | S | 55 | 55 | Decline of vision | Y | 39168 | NFPA | Parafalcine | 8500 | NA | Thyroid tumors |
| 16 | F | S | 60 | 60 | Decline of vision | N | 18848 | NFPA | Parafalcine | 280 | NA | NA |
| 17 | M | S | 62 | 62 | Decline of vision | Y | 17226 | NFPA | Parafalcine | 1320 | NA | NA |
| 18 | F | S | 54 | 54 | Amenorrhea | Y | 10120 | PRL | Tentorium cerebelli | 7524 | NA | NA |
| 19 | F | S | 71 | 71 | Decline of vision | N | 16720 | NFPA | Parafalcine | 4352 | NA | NA |
| 20 | F | P | 66 | 72 | Decline of vision | Y | 47250 | NFPA | T9-10 | 2880 | Transitional | NA |
| 21 | F | S | 70 | 70 | Decline of vision | N | 14175 | NFPA | Left frontal | 1134 | NA | NA |
| 22 | F | S | 50 | 50 | Physical findings | N | 5148 | NFPA | Left CPA | 60000 | Fibrous | NA |
| 23 | F | Men | 66 | 53 | Decline of vision | N | 4704 | NFPA | Left temporal | NA | Transitional | Intraductal papilloma of breast |
| 24 | M | S | 61 | 61 | Physical findings | N | 2925 | NFPA | Parafalcine | 1080 | NA | NA |
| 25 | F | P | 22 | 26 | Amenorrhea | Y | 40500 | NFPA | Right frontal | 20000 | NA | NA |
| 26 | F | S | 52 | 52 | Physical findings | N | 2704 | PRL | Parafalcine | 10080 | Transitional | NA |
| 27 | M | S | 59 | 59 | Decline of vision | Y | 28152 | NFPA | Parafalcine | 150 | NA | NA |
| 28 | F | S | 51 | 51 | Headache | N | 1200 | NFPA | Left frontal | 125 | NA | NA |
| 29 | F | S | 53 | 53 | Physical findings | N | 11475 | NFPA | Right frontal | 3744 | NA | NA |
| 30 | M | S | 49 | 49 | Physical findings | N | 210 | GH | Left sphenoid ridge | 5280 | Transitional | NA |
| 31 | M | S | 57 | 57 | Acromegaly | N | 800 | GH | Tentorium cerebelli | 280 | NA | NA |
| 32 | F | S | 65 | 65 | Headache | N | 2600 | NFPA | Left frontal | 1960 | NA | NA |
| 33 | F | S | 54 | 54 | Decline of vision | N | 15708 | NFPA | Parafalcine | 3780 | NA | NA |
| 34 | F | S | 57 | 57 | PA recurrence | Y | 20000 | NFPA | Parafalcine | 2160 | NA | NA |
| 35 | F | S | 57 | 57 | Physical findings | N | 2856 | NFPA | Left CPA | 29512 | Fibrous | NA |
| 36 | F | S | 67 | 67 | Decline of vision | N | 19320 | NFPA | Right sphenoid ridge | 2448 | NA | NA |
| 37 | F | S | 57 | 57 | Acromegaly | N | 210 | GH | Parafalcine | 1200 | NA | NA |
| 38 | M | S | 65 | 65 | Physical findings | N | 5814 | NFPA | Left temporal | 500 | NA | NA |
| 39 | F | S | 58 | 58 | Decline of vision | N | 20592 | NFPA | Torcular | 1560 | NA | NA |
| 40 | F | S | 56 | 56 | Decline of vision | Y | 4860 | NFPA | Parafalcine | 448 | NA | NA |
| 41 | M | S | 50 | 50 | Acromegaly | Y | 58695 | GH+PRL | Tentorium cerebelli | 15428 | NA | NA |
| 42 | F | S | 56 | 56 | Decline of vision | N | 12000 | NFPA | Right frontal | 2160 | Transitional | NA |
| 43 | F | S | 56 | 56 | Decline of vision | N | 6912 | NFPA | Right frontal | 1859 | Fibrous | NA |
| 44 | M | S | 61 | 61 | Headache | N | 11960 | NFPA | Right frontal | 4056 | Endothelial | NA |
| 45 | F | S | 64 | 64 | Fatigue | Y | 70448 | NFPA | Left frontal,Parafalcine | 62197 | Transitional | Adrenal cortical adenoma |
| 46 | F | S | 56 | 56 | Decline of vision | N | 18000 | NFPA | Right frontal | 6000 | Fibrous | NA |
| 47 | F | S | 57 | 57 | Decline of vision | NA | NA | NFPA | Right frontal | NA | Transitional | NA |
| 48 | F | S | 55 | 55 | PA recurrence | Y | 81120 | NFPA | Left CPA | 8073 | NA | NA |
| 49 | F | S | 61 | 61 | Decline of vision | N | 2431 | NFPA | Left frontal | 3640 | NA | NA |
| 50 | M | S | 59 | 59 | Acromegaly | Y | 13662 | GH | Anterior cranial base | 244818 | Transitional | NA |
| 51 | M | S | 53 | 53 | Headache | N | 510 | NFPA | Right CPA | 3295.08 | NA | NA |
| 52 | F | S | 53 | 53 | Acromegaly | NA | NA | GH | Left parasagittal | 512 | NA | NA |
| 53 | M | S | 54 | 54 | PA recurrence | Y | 8892 | NFPA | Left tentorium cerebelli | 648 | NA | NA |
| 54 | F | S | 49 | 49 | Decline of vision | Y | 30056 | NFPA | Right ventricle; Parafalcine | 19683 | NA | NA |
| 55 | F | S | 68 | 68 | Headache | N | 2496 | NFPA | Left CPA | 29700 | NA | NA |
| 56 | F | S | 57 | 57 | Decline of vision | Y | 10450 | NFPA | Right frontal | 840 | NA | NA |
| 57 | F | S | 57 | 57 | Visual field defects | Y | 3876 | NFPA | Right orbital | 2940 | NA | NA |

**Abbreviation:** M, male, F, female, Y, yes, N, no, NA, not available, S, Simultaneous, P, pituitary adenoma, Men, meningioma, NFPA, non functional pituitary adenoma, CPA, Cerebellopontine angle, GH, [growth hormone](http://www.so.com/link?url=http%3A%2F%2Fdict.youdao.com%2Fsearch%3Fq%3Dgrowth%2520hormone%26keyfrom%3Dhao360&q=%E7%94%9F%E9%95%BF%E6%BF%80%E7%B4%A0+%E7%BF%BB%E8%AF%91&ts=1528797531&t=284ecce2c1c8a3cde3f249e1b263a56) , PRL, prolactin.
